# Hypothalamic Agouti‐Related Peptide mRNA is Elevated During Natural and Stress‐Induced Anorexia

**DOI:** 10.1111/jne.12295

**Published:** 2015-08-20

**Authors:** I. C. Dunn, P. W. Wilson, R. B. D'Eath, T. Boswell

**Affiliations:** ^1^ The Roslin InstituteRoyal (Dick) School of Veterinary StudiesUniversity of EdinburghEdinburghUK; ^2^ Animal Behaviour & WelfareVeterinary Science Research GroupSRUCWest Mains RoadEdinburghEH9 3JGUK; ^3^ School of BiologyCentre for Behaviour and EvolutionNewcastle UniversityNewcastle‐Upon‐TyneUK

**Keywords:** broodiness, maternal behaviour, stress, energy balance

## Abstract

As part of their natural lives, animals can undergo periods of voluntarily reduced food intake and body weight (i.e. animal anorexias) that are beneficial for survival or breeding, such as during territorial behaviour, hibernation, migration and incubation of eggs. For incubation, a change in the defended level of body weight or ‘sliding set point’ appears to be involved, although the neural mechanisms reponsible for this are unknown. We investigated how neuropeptide gene expression in the arcuate nucleus of the domestic chicken responded to a 60–70% voluntary reduction in food intake measured both after incubation and after an environmental stressor involving transfer to unfamiliar housing. We hypothesised that gene expression would not change in these circumstances because the reduced food intake and body weight represented a defended level in birds with free access to food. Unexpectedly, we observed increased gene expression of the orexigenic peptide agouti‐related peptide (AgRP) in both incubating and transferred animals compared to controls. Also pro‐opiomelanocortin (POMC) mRNA was higher in incubating hens and significantly increased 6 days after exposure to the stressor. Conversely expression of neuropeptide Y and cocaine‐ and amphetamine‐regulated transcript gene was unchanged in both experimental situations. We conclude that AgRP expression remains sensitive to the level of energy stores during natural anorexias, which is of adaptive advantage, although its normal orexigenic effects are over‐ridden by inhibitory signals. In the case of stress‐induced anorexia, increased POMC may contribute to this inhibitory role, whereas, for incubation, reduced feeding may also be associated with increased expression in the hypothalamus of the anorexigenic peptide vasoactive intestinal peptide.

Vertebrate animals change their feeding behaviour to balance the energetic demands of physiological processes (e.g. reproduction) with environmental conditions and many species show seasonal cycles in food intake and fat deposition. Many rodents, for example, undergo increased appetite and fattening during long days in summer 1, acting in some species as preparation for hibernation 2. Similarly, migratory birds increase food intake and deposit fat as fuel for migratory flight 3. Conversely, a voluntary reduction of feeding may occur in situations where other annual cycle events temporarily have greater value for fitness than feeding. Examples of such animal anorexias 4, when individuals eat little in the presence of food, or fail to forage for food, can be observed in some species during hibernation, migration, defence of a territory or harem, moulting and incubation behaviour. The suppression of feeding avoids conflicts with these activities and is therefore generally adaptive. However, in livestock, reduced food intake (e.g. during disease, inadequate nutrition or induced by stress) represents a challenge to animal production 5. Similarly, reduced food intake is of human medical importance in relation to anorexia nervosa and other eating disorders, as well as loss of appetite during disease (cachexia) 6.

The characterisation of leptin and its receptors led to the identification of neural circuitry in the arcuate nucleus that coordinates the compensatory behavioural and physiological responses to the loss of energy stores induced, for example, by fasting or food restriction 7. One arcuate nucleus cell group exerts an anabolic effect through expression of agouti‐related peptide (AgRP) and neuropeptide Y (NPY) and reciprocally innervates a second cell group that induces an anabolic influence via expression of the pro‐opiomelanocortin (POMC) and cocaine‐ and amphetamine‐regulated transcript (CART) genes. Although first identified in laboratory rodents, the arcuate nucleus is neuro‐anatomically and functionally conserved in relation to energy balance regulation in other vertebrates, such as fish 8 and birds 9, 10. As in mammals, the peptide products of the AgRP and POMC genes exert their effects through the melanocortin 4 receptor (MC4R) in birds 11, 12 which is well conserved across species. The action of arcuate nucleus neuropeptides contributes to the homeostatic regulation of body weight around a defended level that may be adjusted seasonally, by defence of a ‘sliding set point’ or ‘rheostasis’ 13. However, the mechanisms involved in such seasonal body weight regulation are unclear. Several studies have investigated whether seasonal changes in appetite and fat deposition in mammals such as Siberian hamsters and sheep are driven by correlated changes in expression of anabolic and catabolic arcuate nucleus neuropeptide genes 14, 15, 16. However, the changes in gene expression observed have generally not been consistent and, in Siberian hamsters, chemical ablation of the arcuate nucleus or experimental over‐expression of AgRP was not found to disrupt seasonal body weight cycles 17, 18. Instead, more recent evidence suggests that the regulation of seasonal body weight cycles may involve changes in gene expression in the ependymal cells lining the third ventricle under the control of thyroid hormones 19.

There have been limited investigations into the neuroendocrine mechanisms regulating a voluntary reduction in food intake beyond the general context of seasonal cycles in body weight and appetite in mammals. The domestic chicken is a valuable model for such investigations because several strains show the ancestral characteristic of a naturally‐occurring anorexia during incubation of their eggs. Over a 3‐week period, hens display ‘broody’ behaviour 20, directing their activity towards sitting on the nest and spending little time feeding, which results in a loss of body weight despite food being readily available 21, 22. The neuroendocrine basis for the maintenance of avian incubation behaviour is the secretion of prolactin from the pituitary in response to vasoactive intestinal peptide (VIP) released from neurones in the basal hypothalamus that project to the median eminence 23. We recently investigated the homeostatic regulation of body weight by the arcuate nucleus in chickens. We demonstrated, in broiler (meat‐producing) chickens, that AgRP and NPY expression was increased by food restriction and that AgRP gene expression differed between birds at the same body weight but with a different recent history of food availability 24. By contrast, POMC gene expression was unaffected by food restriction. These observations are consistent with a role for AgRP in the homeostatic regulation of body weight in chickens. The present study aimed to extend our investigation into two situations of a voluntary reduction of food intake in chickens. The first of these was during incubation, in which anorexia has been linked to a sliding set point for body weight 4. The second situation was the effect of stress imposed by relocating laying hens from cages into group housing, which we had previously observed to be accompanied by a reduction in food intake in the days following the move. Reduced food intake was not imposed on the birds in either case, unlike the situation with food restriction. For incubation, we predicted that, if a reduced set point for food intake was involved during the phase of weight loss, we would not observe increased hypothalamic AgRP and NPY gene expression, concomitant with a reduced drive to eat. The stress‐induced reduction of feeding offers a useful comparison because it has not been linked to a reduced set‐point for appetite. Relatively little is known about the role of the melanocortin system and other arcuate nucleus neuropeptides in the regulation of stress‐induced anorexia compared to corticotrophin‐releasing factor and other adrenocorticotrophic hormone‐releasing peptides. However, stress‐induced anorexia was reversed by central melanocortin 4 receptor blockade in rats, suggesting that the brain melanocortin system is involved 25.

Overall, we hypothesised that AgRP levels might remain low or unchanged during both incubation and cage‐to‐pen transfer associated with a reduced drive to eat. Unexpectedly, in both situations, AgRP mRNA levels in the basal hypothalamus were significantly greater compared to control animals. POMC was also significantly increased during stress‐induced anorexia.

## Materials and methods

### Animals

Experiments were performed using the female offspring (hens) of an F1 cross between female white leghorns and male silkie chickens, which have a high propensity to express natural incubation behaviour 26.

Animal experiments were performed according to UK Home Office legislation under project license PPL 60/3964 and the hens were killed by overdose of i.v. pentobarbitone, a schedule 1 method.

#### Experiment 1: Incubating versus laying hens

Laying hens (20–24 weeks of age) were ranked and randomised on the basis of body weight into two treatment groups and housed in individual pens. The objective was to compare two treatment groups (n = 12 each): (i) incubating hens (INC), comprising an ‘incubation’ treatment where laying hens were provided with the appropriate environment of a nest box, nesting material and a clutch of eggs to allow incubation behaviour to develop and (ii) laying control (LC) hens, comprising a ‘non‐incubating control’ treatment where laying hens were not allowed the appropriate environment for incubation behaviour to develop. An identical nest box was present but upturned to prevent entry and the pen was otherwise identical. Food was available *ad lib*. in all pens at all times and intake was monitored daily by weigh‐back where a known weight of food is present at the start and the food is weighed each day to estimate the amount consumed. A voluntary reduction in food intake was expected in the INC group. A LC hen was recruited to the study when the INC hen started to incubate. Therefore, hens were taken through the study as a block of two (INC and LC) and killed at the same time. Birds were examined daily for the outward signs of incubation: sitting, aggression and distinctive vocalisation.

All birds were killed 21 days after INC hens had started incubation and samples of basal hypothalamus were taken as described previously 24, 27.

#### Experiment 2: Incubating versus pair‐fed hens and pair‐fed hens released from restriction

The paradigm of Experiment 1 was repeated with a new set of hens ranked and randomised by body weight. However, in light of the experience of the first experiment, the design was modified to include pair‐fed hens to contrast with the voluntary food restriction of the incubating hens and to use a further control group that was released from the pair‐fed restriction. A block of three hens from each of three treatment groups was formed each time an incubating hen was identified. There were three treatment groups (n = 12 each). (i) INC, comprising incubation as in Experiment 1. Hens were allowed *ad lib*. access to food and food intake was recorded. (ii) Pair‐fed control (PFC), comprising a control treatment where hens were not allowed the appropriate environment for incubation, preventing the behaviour and provided with rationed food matched to that consumed by birds in the INC treatment (pair‐fed). These birds thus underwent similar but involuntary food restriction. Food intake was averaged over 3 days to derive the pair‐fed level, and so there was a delay in the onset of the reduction in food intake between INC and PFC, which were fed to the same level as the INC bird. After 16 days, the food restriction was maintained on a plane determined by that of the mean on the sixteenth day by which time birds were no longer laying eggs. (iii) PFC Release, comprising a second PFC group similar to the PFC but released from restriction after 16 days. All three hens in the block were killed 21 days after the INC bird had commenced incubation behaviour.

This treatment avoiding the potential confounding effect in Experiment 1 of control birds continuing to lay, in contrast to the incubating birds. Therefore, in Experiment 2, all groups were not laying eggs so that we were able to contrast directly a voluntary (INC) and an involuntary reduction (PFC) in food intake with a hen with free access to food (PFC Release).

#### Experiment 3: Cage‐to‐pen transfer stress

When setting up Experiment 1, it was noted that, after transferring birds from single housing in a cage to an individual pen, the hens showed a pronounced natural anorexia, reducing their food intake to 30% of the control hens, presumably related to the stress of transfer and a new environment. This resulted ultimately in many birds temporarily ceasing laying, which delayed the start of the incubation experiments. We made use of this serendipitous observation to carry out an additional study. For this experiment, we killed hens 6 days after transfer along with birds from a control group that remained in cages (n = 8). Food intake was recorded in both environments by weigh‐back.

### Measurement of gene expression

All tissue sampling and processing were carried out as described previously 24. Birds were killed and the basal hypothalamus, pre‐optic area of the brain and pituitary were snap‐frozen in liquid nitrogen. AgRP, NPY and POMC and CART mRNA levels were quantified in the basal hypothalamus using a real‐time polymerase chain reaction (PCR) as described previously 24. The real‐time PCR assay for VIP mRNA levels was a new assay; however, the methodology was identical to that described for the genes above. Primers VIPpw1F (GATGCAGCCAGTGAATCTGA) and VIPpw1R (GAGTGGCGTTTGACAGGACT) were used that amplified a product of 149 bp over an intron–exon boundary. The product identity was confirmed by sequencing. As employed previously, the lamin B receptor (Lbr) gene was used as a housekeeping gene to normalise expression 24, 28.

### Corticosterone assay

Plasma corticosterone concentrations were measured using a Corticosterone EIA Kit ADI‐901‐097 (Enzo Life Sciences, Farmingdale, NY, USA). The assay was validated for chicken exactly in accordance with the protocol laid out for white crowned sparrows 29. Briefly, the assay relies on a steroid displacement reagent to ensure total corticosterone is measured when validation utilises increasing dilutions (1 : 10, 1 : 20, 1 : 40 and 1 : 60) of stripped plasma (1% Norit A charcoal and 0.1% dextran in water) spiked with a known amount of corticosterone (500 pg/ml). The steroid displacement reagent was assessed at 0%, 1% and 2% of plasma volume. It was found that plasma dilutions between 1 : 20 and 1 : 40 combined with 2% steroid displacement reagent gave the best recovery. A further validation determined that a 1 : 20 dilution of plasma was optimal to ensure that all samples from hens with high corticosterone were in the range of the standard curve and, subsequently, all samples were assayed at that dilution in a single assay.

The accuracy of the assay as defined by percent recovery of a known amount of corticosterone was 90.7%. Linearity of diluted plasma was R^2^ = 0.94 and was parallel to the standard curve material as supplied in the assay kit. The lower limit of detection was 48.75 pg/ml as determined by the concentration of corticosterone measured at two SDs from the zero along the standard curve. Inter‐ and intra‐assay coefficients of variation were 13.62% and 7.87%, respectively. All quality control samples and plasma samples were stored at −20 °C and assayed at first thaw.

### Statistical analysis

Results were analysed by anova in Genstat, version 13 (VSN International, Hemel Hempstead, UK) using log transformed data to approximate to a normal distribution where appropriate. In Experiments 1 and 2, the hen pair or triplet blocks were treated as fixed effects. An exception was the analysis of follicle numbers in Experiment 1, which was performed using a Kruskal–Wallis test because the data distribution was not suitable for anova.

## Results

### Experiment 1: Incubating versus laying hens

In INC hens, voluntary anorexia was indicated by a 23% reduction in body weight over the 21‐day period, reflecting a loss of approximately 300 g at post mortem compared to a laying hen (Table 1). As expected, the INC hens had regressed reproductive systems with no yellow yolky follicles, smaller ovaries and an approximately 10‐fold reduction in oviduct weight compared to laying hens (Table 1). The pituitary was significantly larger in the INC hens but no difference was observed in the adrenal or spleen tissue weights (Table 1).

**Table 1:** Body and Organ Weights and Yellow Yolky Follicles Numbers (YYF) in Incubating and Laying Hens

Tissue	Laying	Incubating	P^a^
Body weight (g)	1431 ± 26	1127 ± 21	< 0.001
Pituitary (mg)	7.9 ± 0.5	10.2 ± 0.8	0.016
Ovary (g)	6.5 ± 0.4	1.8 ± 0.1	< 0.001
Oviduct (g)	39.9 ± 2.2	3.3 ± 0.5	< 0.001
Spleen (g)	1.9 ± 0.2	1.8 ± 0.1	0.712
Adrenal (mg)	112.8 ± 9.7	98.8 ± 6.2	0.278
Number of YYF^b^	5.6 ± 0.2	0	< 0.001

^a^Significance from anova (F_1,11_). ^b^Kruskal–Wallis anova (n = 12; mean ± SEM).

**Figure 1:**
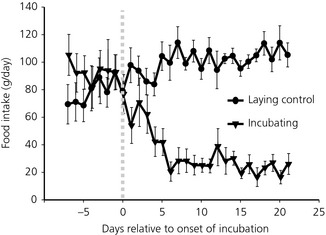
Daily food intake of hens plotted in relation to when a bird was judged to be showing incubation behaviour. The grey dotted line indicates where incubation behaviour was recognised. The laying control hens were paired to an incubating hen at the time of the onset of incubation behaviour (n = 12; mean ± SEM).

Food intake decreased dramatically from around 100 g per hen per day prior to the onset of incubation behaviour to approximately 20 g per hen per day when incubation behaviour was fully established (Fig. 1).

**Figure 2:**
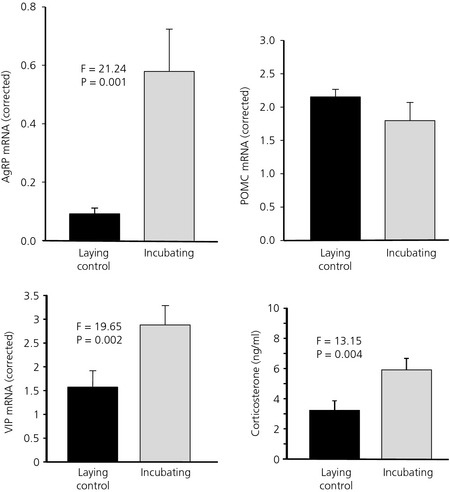
Agouti‐related peptide (AgRP), pro‐opiomelanocortin (POMC) and vasoactive intestinal peptide (VIP) mRNA in the basal hypothalamus and circulating corticosterone levels of incubating and laying control hens. Values were corrected using lamin B receptor (Lbr) gene expression (n = 11; mean ± SEM).

Basal hypothalamic AgRP mRNA was approximately five‐fold higher in INC hens and lowest in controls (LC) (F_1,9_ = 21.24, P = 0.001). There was no difference in the expression of POMC mRNA (Fig. 2). We also measured expression of the NPY and CART genes, co‐expressed in AgRP and POMC neurones, respectively, which are also implicated in orexigenic and anorectic actions. Neither NPY (F_1,9_ = 0.23, P = 0.64), nor CART (F_1,9_ = 0.26, P = 0.62) mRNA was significantly different between the LC or INC. Finally, VIP, the prolactin releasing factor, was expressed at approximately twice the level in the hypothalamus of INC compared to the LC hens (F_1,9_ = 19.65, P = 0.002) (Fig. 2).

When the levels of pituitary POMC mRNA (as a precursor of adrenocorticotrophic hormone) were examined, there was a statistical difference with the INC hens having lower expression. However, when corrected for the differences as a result of the significantly greater amount of total RNA extracted (40.1 ± 4.8 versus 20.1 ± 4.2 μg, P = 0.015) from the larger INC hen pituitary (10.2 ± 0.8 versus 7.9 ± 0.5 mg, P = 0.016) the levels per pituitary were not different. However, circulating corticosterone concentrations were higher (F_1,11_ = 13.15, P = 0.004) in INC hens than in the LC hens (Fig. 2).

### Experiment 2: Incubating versus pair‐fed hens and pair‐fed hens released from restriction

**Figure 3:**
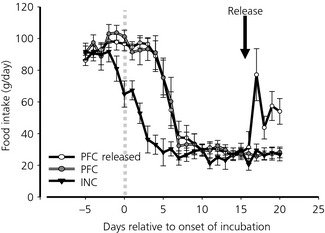
Food intake in incubating (INC), pair‐fed control (PFC) and pair‐fed control hens released from restriction (PFC released). The grey dotted line indicates where incubation behaviour was recognised and the data for pair feeding began to be collected. The black arrow indicates when the pair‐fed control released hens were released from restriction (n = 12; mean ± SEM).

Having established the model of naturally reduced food intake and changes in hypothalamic expression of AgRP in Experiment 1, we then compared hens that were pair‐fed to the same level as the INC hens and hens treated the same but released from restriction. As in Experiment 1, the hens reduced their food intake when they started incubating. There was a lag between the application of the pair‐fed restriction and the decrease in food intake of the INC hens (Fig. 3) but a similar decrease in food intake was achieved within 7 days and the body weights (Table 2) reached were not statistically different between the INC and PFC hens at the end of the experiment. Food intake increased rapidly after release from restriction but was lower than prior to the onset of incubation.

**Table 2:** Body and Organ Weight and Yellow Yolky Follicles Numbers (YYF) in Incubating, Pair‐Fed Control and Pair‐Fed Control Released from Restriction

Tissue	Pair fed released	Incubating	Pair fed	P^a^	LSD^b^
Body weight (g)	1194 ± 27	1073 ± 21	1096 ± 28	0.008	69.8
Pituitary (mg)	4.9 ± 0.4	7.4 ± 0.8	4.4 ± 0.3	< 0.001	1.5
Ovary (g)	2.3 ± 0.2	1.6 ± 0.1	2.0 ± 0.2	0.123	
Oviduct (g)	5.0 ± 0.4	2.7 ± 0.1	5.3 ± 1.6	0.273	
Spleen (g)	2.3 ± 0.2	1.6 ± 0.1	2.2 ± 0.4	0.233	
Adrenal (mg)	89 ± 7.9	106.3 ± 5.7	113.5 ± 9.2	0.076	
Number of YYF	0	0	0.3		

The P value for the analysis of variance^a^ (F_2,21_) and the least significant difference (LSD) at P = 0.05^b^ is presented where appropriate to allow comparison between groups. (n = 12; mean ± SEM).

There was a statistical difference in body weight between the three groups, with the PFC Released hens being approximately 100 g heavier (P < 0.001) than the birds remaining on voluntary restriction (INC) or involuntary restriction (PFC) (Table 2). Of the other organs measured, only the pituitary showed statistical significant differences in its weight, with the INC hens having a heavier pituitary (> 50%) than the pair‐fed groups regardless of whether released or not from restriction. All the measures indicated that all groups had regressed reproductive tracts with either no or almost no yellow yolky follicles (one PFC bird still had four follicles) and small oviducts (Table 2). There was tendency for the hens released from restriction (PFC Released) to have a smaller adrenal weight. However, in this experiment, there was no statistical difference in plasma corticosterone concentrations, although the INC hens did have higher titres than the PFC or PFC released groups (PFC Released, 4.63 ± 1.02 ng/ml; INC 9.04 ± 2.79 ng/ml; PFC, 5.51 ± 1.3 ng/ml).

**Figure 4:**
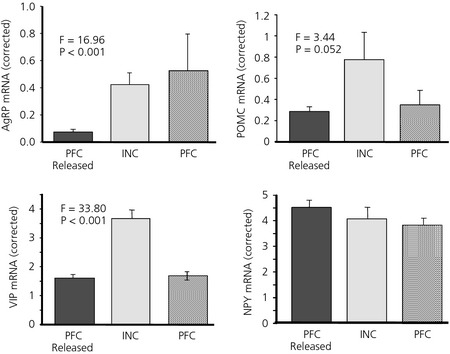
Agouti‐related peptide (AgRP), pro‐opiomelanocortin (POMC), vasoactive intestinal peptide (VIP) and neuropeptide Y (NPY) mRNA expression in the basal hypothalamus of incubating (INC), pair‐fed control (PFC) and pair‐fed control released from restriction (PFC Released) hens. Values were corrected using Lbr gene expression (n = 12; mean ± SEM, F_2,21_).

There were highly significant differences between the groups for expression of AgRP in the basal hypothalamus (anova, F_2,21_ = 16.96, P < 0.001). The AgRP levels in the INC hens compared to those feeding *ad libitum* (PFC Released) (Fig. 4) were higher (P < 0.001). The pair‐fed hens undergoing involuntary food restriction (PFC) had an expression of AgRP mRNA in the hypothalamus similar to the INC hens that had voluntarily reduced their food intake to the same level (Fig. 4).

The anova on the basal hypothalamic POMC mRNA levels indicated that there was a near significant effect of treatment (F_2,20_ = 3.32, P = 0.052). Comparison between the voluntary anorexia (INC) and either of the groups of hens that were either involuntarily restricted (PFC) or released from restriction (PFC Released) revealed higher levels of POMC mRNA in INC hens (F_2,20_ = 3.44, P = 0.052). anova indicated that neither NPY (F_2,21_ = 0.76, P = 0.478) (Fig. 4), nor CART (F_2,21_ = 0.68, P = 0.516) was significantly different. VIP levels were significantly different (F_2,21_ = 33.80, P < 0.001) (Fig. 4), with over twice the level of mRNA in the hypothalamus of INC hens compared to either of the pair‐fed groups (P < 0.001) (Fig. 4).

### Experiment 3: Cage‐to‐pen transfer stress

**Figure 5:**
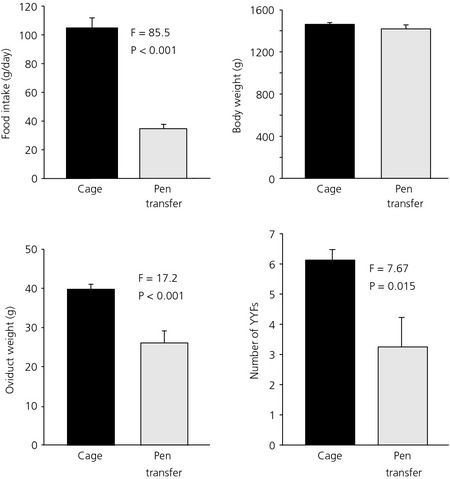
Food intake, body and oviduct weight and number of yellow yolky follicles (YYF) in the ovary of hens kept in cages or transferred to pens (mean ± SEM; n = 8, F_1,14_).

In the cage‐to‐pen transfer, a 70% reduction in food intake was recorded over the experimental period, although there was no statistically significant difference from LC hens in body weight (Fig. 5). The ovaries of the cage‐to‐pen‐transferred birds had started to regress compared to the LC hens, which remained in cages, as indicated by the reduced number of yellow yolky follicles (Fig. 5). The reduction in oviduct weight observed in the transferred hens is a downstream consequence of this (Fig. 5).

**Figure 6:**
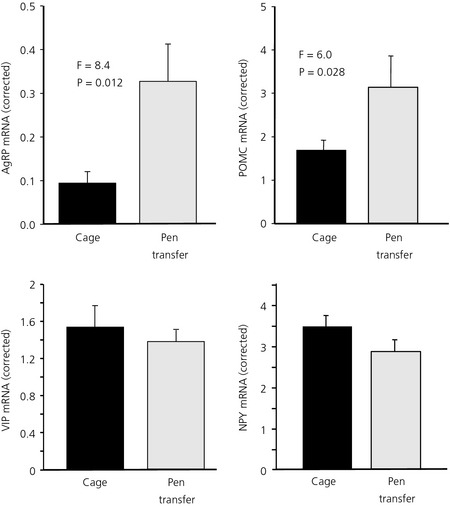
Agouti‐related peptide (AgRP), pro‐opiomelanocortin (POMC), vasoactive intestinal peptide (VIP) and neuropeptide Y (NPY) mRNA expression in hens kept in cages or transferred to pens. Values were corrected using Lbr gene expression (mean ± SEM; n = 8, F_1,14_).

AgRP mRNA expression in the basal hypothalamus was three‐ to four‐fold higher in the transferred hens (F_1,14_ = 8.4, P = 0.012) combined with a higher expression of POMC mRNA (F_1,14_ = 6.0, P = 0.028) (Fig. 6). NPY (F_1,14_ = 1.03, P = 0.328) and CART (F_1,14_ = 1.12, P = 0.308), and the expression of VIP mRNA (F_1,14_ = 0.22, P = 0.648) levels in the basal hypothalamus was not different between the two groups (Fig. 6).

Corticosterone levels were not significantly different between transferred birds and LC hens (anova, F_1,14_ = 1.52).

## Discussion

We have investigated two examples of natural avian anorexias: one comprising the incubation behaviour model investigated by Sherry and Mrosovsky 4, 13, 22 and the other that was induced by a stressful event. For incubation, we hypothesised that, if body weight is regulated by a sliding set point, and that the role of arcuate nucleus peptides is solely to restore the defended level of body weight after perturbation, then no change in gene expression would be expected when food is freely available to INC hens because body weight is at the defended level. By contrast, in PFC hens, the gene expression of orexigenic peptides would be expected to increase as a result of negative energy balance being imposed upon the birds. Unexpectedly, although AgRP expression was increased in PFC hens as predicted, it was also elevated in INC hens. A similar finding was obtained after transferring hens from cages into pens: AgRP expression was increased compared to birds kept in cages, despite the fact that food intake was significantly decreased when being available *ad lib*. Thus, in both situations, the anabolic drive provided by increased AgRP signalling must be over‐ridden by others factors. For cage‐to‐pen transfer, naturally reduced food intake was associated with increased POMC expression, which may contribute to the anorectic drive. Increased POMC mRNA was only marginally significant in the incubation experiment but a negative influence could be provided by the increase in hypothalamic VIP gene expression that is classically associated with incubation.

Earlier studies of the INC hen model have focused on comparisons between laying and INC hens 21, 22. The 80% reduction in food intake that we observed is comparable to that previously observed. However, directly comparing laying and INC hens is complicated by the fact that the reduced food intake and body weight can be at least partly attributed to regression of the reproductive system. This means that the resulting reduction in ovarian steroids can make interpretation of physiological changes more difficult. However, in our experiments, and as noted previously 21, 22, the reduction in body weight is greater than simply that measured in the weight of the reproductive organs. Also, in Experiment 2, we removed the confounding effect of reproductive status by comparing INC hens with PFC hens. Food restriction imposed by pair‐feeding resulted in ovarian regression presumably as a result of reduced gonadotrophin‐releasing hormone hypothalamic drive and plasma gonadotrophin concentrations as observed in previous studies 30. When one of a pair‐fed group was released from restriction, we observed that food intake was significantly higher than in INC hens but lower than in laying birds. Importantly, this indicates that food intake is naturally higher in the non‐incubating state independent of the increased metabolic demand of full reproductive development. The comparable regressed state of the reproductive system in all groups in Experiment 2 demonstrates that the changes in gene expression measured cannot be accounted for simply by altered ovarian steroid production.

In both the incubation and cage transfer experiments, AgRP expression was increased in the groups in which food intake was voluntarily reduced. This suggests that AgRP expression is still responsive to a loss of energy stores but the apparent increase in AgRP signalling does not result in increased feeding, presumably because of the presence of over‐riding anorexigenic signals. This observation may be of functional significance during incubation when a hen may need to abandon the nest if energy stores fall too low 4, 22. Similarly, in the case of a stressful event, the fact that AgRP expression continues to reflect nutritional state would allow energy balance to be restored more quickly as the animal adapts to the altered conditions. For cage‐to‐pen transfer and, to a lesser extent, incubation, increased POMC expression may play a role in over‐riding AgRP signalling to cause the reduced food intake. In the limited number of studies where AgRP/NPY or POMC/CART have been measured in mammalian models of stress that result in anorexia, there is some evidence for increased expression of the orexigenic peptide genes but the significance of this finding is uncertain. Concurrent overexpression of both AgRP and POMC during a phase of reduced feeding following exposure to acute restraint stress was recently observed in rats 31, although POMC expression was increased to a relatively larger extent than AgRP. Similar observations were made after restraint and forced‐swim when c‐fos mRNA expression was observed in a high percentage of POMC neurones and in a smaller population of AgRP cells 32. Modest increases in expression of NPY were observed in the arcuate nucleus of rats subject to mild foot shock treatment that induced anorexia over a short time period, although AgRP was significantly reduced and this was linked to a greater sensitivity of the HPA axis to α‐melanocyte‐stimulating hormone 33. The increase in AgRP expression we observed in the present study was of relatively greater magnitude than observed in these rat studies.

Interpretation of the significance of our observations of increased AgRP and POMC gene expression would be aided by knowledge of any corresponding changes in melanocortin receptor expression, which we did not measure in the present study. Previous reports in birds have been limited to the MC4R for which increased gene expression has been observed following food deprivation 34, 35. Changes in constitutive MC4R gene expression could negate any positive or negative effects on food intake implied by the increased mRNA levels that we recorded for AgRP and POMC in our experiments. It was recently demonstrated in mice that AgRP can act independently on MC4R receptors as a biased agonist in addition to its well‐known antagonistic effect 36. This could provide an explanation for the unexpected changes in melanocortin gene expression that we observed (but has not yet been investigated) in birds.

In laboratory rodents, AgRP and POMC gene expression is increased and decreased, respectively, by circulating corticosterone 37, although the situation is less clear in birds 38, 39. We observed no clear relationship between corticosterone concentrations and neuropeptide expression in our experiments. In the incubation studies, corticosterone titres were significantly higher in INC compared to laying birds, although this difference was only apparent as a nonsignificant trend between INC and pair‐fed birds in Experiment 2. In the cage‐to‐pen transfer study, no significant difference in corticosterone concentrations was observed, although it is possible that they had returned to normal levels in the stressed group by the time of sampling, 6 days after the transfer took place. In mammals, arcuate nucleus neuropeptide expression is regulated by leptin but little is known about this in birds because the existence of leptin genes in avian genomes has been historically contentious 40. Leptin genes have now been identified from genome sequencing projects in several avian taxa but not yet in the chicken 41. Also, the tissue distribution of expression of avian leptins studied so far appears centred on the pituitary rather than adipose tissue 42, 43, making the extent to which leptin is a key nutritional signal in birds unclear.

For the incubation experiment, the fact that POMC gene expression was only marginally significantly higher in the INC compared to the pair‐fed groups, suggests that other signals may be responsible for reducing voluntary food intake. VIP is the main candidate because its expression was significantly elevated in INC hens in both incubation experiments and VIP peptide reduces food intake in domestic chicks after central injection 44. The inter‐relationship between VIP neurones and AgRP/NPY neurones is not clear. VIP mRNA and immunoreactive cell bodies have been observed in the chicken arcuate nucleus 45 but it is not known whether VIP and AgRP mRNAs are co‐expressed. The arcuate nucleus is also a site of expression of VIP receptor (VIPR1) mRNA in the turkey (*Meleagris gallopavo*) and was particularly highly expressed in this nucleus during incubation compared to other reproductive states 46. It will therefore be important to determine whether VIPR1 or VIPR2 mRNAs are expressed by AgRP/NPY neurones in the chicken. Alternatively, because VIP is well‐known as the main hypothalamic releasing factor for prolactin in birds 20, 47, reduced food intake may be part of the suite of behavioural and physiological changes regulated by the high circulating prolactin levels associated with incubation. Although prolactin is generally considered to stimulate food intake, its effects in birds have historically been studied only through the administration of mammalian prolactin 48. Interestingly, the only study to investigate feeding effects of recombinant native prolactin in birds reported an inhibitory effect of the hormone on food intake in laying turkey hens after central injection 49, which is consistent with a possible inhibitory effect of prolactin during incubation. However, in ring doves (*Streptopelia risoria*), central administration of mammalian prolactin stimulated feeding and was associated with increased NPY‐immunoreactive cell bodies in the arcuate nucleus 50. Therefore, we cannot rule out a direct stimulatory effect of prolactin for inducing increased AgRP expression in our INC hens. Although AgRP expression may be influenced by VIP and/or prolactin directly, there is also evidence to suggest that VIP reduces food intake in chickens independently of the melanocortin system. Central injection of both mammalian and chicken VIP peptides 50, 51 appears to decrease food intake in the chicken via the HPA axis. For example, central injection of chicken VIP in domestic chicks led to increases in plasma corticosterone and diencephalic expression of corticotrophin‐releasing hormone (CRH) and CRH receptor 2 (CRHR2), and the inhibitory effect of central VIP administration on food intake was partly ameliorated by the CRHR2 antagonist astressin 51. Our observation of significantly increased plasma corticosterone in INC hens compared to LC hens supports the possibility that the reduced food intake shown by INC hens is mediated through hypothalamic CRH.

By contrast to AgRP and POMC, we observed no consistent changes in the expression of the other arcuate nucleus neuropeptides CART and NPY. CART gene expression did not change in any of our experiments, in line with previous observations after manipulations of energy balance in birds 24, 35. Thus, although CART peptide inhibits food intake after central injection in domestic chicks 44, its significance in birds in the regulation of energy balance remains uncertain. For NPY, which is known to be co‐expressed in AgRP neurones in birds as in mammals 9, we would have expected the patterns of gene expression in our experiments to parallel those of AgRP as observed previously 24. However, we observed no significant changes in NPY mRNA either during incubation or after cage‐to‐pen transfer. This may indicate differential regulation of AgRP and NPY expression in these circumstances. However, our dissection is more variable for NPY because, unlike the case for AgRP, POMC and CART where expression is limited to a discrete set of neurones within the arcuate nucleus, it samples additional NPY cell groups in the surrounding area. This may contribute to more variability, making statistical differences in gene expression harder to detect, although we were able to differentiate birds at different levels of food restriction from their NPY mRNA levels 21. The unaltered gene expression of NPY and CART in our models of natural anorexia is actually in line with our original prediction that arcuate nucleus neuropeptide expression would not change because food intake and body weight are at the defended level. Similar findings of either unaltered gene expression, or paradoxical expression in relation to the behavioural effects of the encoded peptides, have been common in investigations studying seasonal voluntary food intake and body weight cycles in mammals 19. Here, photoperiodically‐driven seasonal cyclicity has been linked to distinct patterns of gene expression in glial cells, particularly ependymal cells, coordinated by local production and action of thyroid hormones 19. However, the pathways through which gene expression in glial cells leads to seasonal rheostasis in appetite and body weight are currently unknown and it is uncertain whether the same rheostatic mechanisms apply to seasonal weight cycles that are directly driven by photoperiod compared to reduced feeding during incubation, which is expressed as part of a suite of changes comprising parental behaviour.

Our observations of increased AgRP gene expression during anorexia are reminiscent of findings of studies of the effects of parasitic infection in rats and fish 52, 53, 54. In rats, NPY gene expression in the arcuate nucleus was increased 8 days after infection, coincident with decreased food intake 52. In rainbow trout (*Oncorhynchus mykiss*), reduced food intake 4 weeks after infection was accompanied by significantly increased AgRP and POMC mRNA but reduced NPY mRNA 54. Leptin was investigated as a possible causative agent for the anorexia in both species. In the rainbow trout, plasma leptin‐A1 concentrations were significantly increased in infected individuals linked to an effect of the parasite in inducing hypoxia, a known stimulant of leptin secretion in fish. The direction of changes in hypothalamic gene expression in infected fish was consistent with previously reported effects of leptin administration 54. In rats, there was a tendency for leptin to be high in some individuals during the early stages of infection but the anorexia appeared to be maintained by other unknown signals produced by the presence of the parasite 53. Given that leptin is still apparently absent from the chicken genome, as noted above, it is difficult to draw parallels at the regulatory level between these models of parasite‐induced anorexia and our own findings.

Although knowledge of the phenomenon of a voluntary reduction of food intake and body weight in INC hens, as well as experimental evidence that it is homeostatically defended, has been available for more than 35 years, we currently know little about how it is regulated at the molecular level. Our findings on AgRP expression suggest that its responsiveness to a loss of energy stores is maintained during incubation and after a cage‐to‐pen transfer in the face of a voluntary reduction in food intake when food is freely available. This may be of functional significance in allowing INC hens to monitor their energy stores and perhaps to feed vigorously when they do leave the nest, and for the process of recovery following a stressful event. However, the key factors driving the voluntary loss of appetite and body weight remain to be unambiguously confirmed experimentally.
